# Dietary Anthocyanins and Human Health

**DOI:** 10.3390/nu11092107

**Published:** 2019-09-05

**Authors:** Christopher N. Blesso

**Affiliations:** Department of Nutritional Sciences, University of Connecticut, Storrs, CT 06269, USA; christopher.blesso@uconn.edu

**Keywords:** anthocyanins, flavonoids, berries, polyphenols, inflammation, bone, gut health, gut microbiota, cardiovascular disease, metabolic syndrome

## Abstract

Anthocyanins may contribute to the inverse relationship between fruit and vegetable intake and chronic disease. Anthocyanins are pigments found in plant structures that consist of an anthocyanidin (aglycone) attached to sugar moieties. Anthocyanins may be beneficial for health through effects on cellular antioxidant status and inflammation; however, their underlying mechanisms of action in their protection of chronic diseases are likely complex and require further elucidation. This Special Issue comprises 8 peer-reviewed papers (including 6 original research articles) which highlight the diverse bioactivities of anthocyanins and anthocyanin-rich foods in the protection against chronic disease.

Plants supply a significant amount of anthocyanins (ACNs) to the human diet, which are thought to contribute to the inverse relationship between fruit and vegetable intake and chronic disease [1]. ACNs are pigments found in plant structures that consist of an anthocyanidin (aglycone) attached to sugar moieties [2]. Cyanidin, peonidin, pelargonidin, malvidin, delphinidin, and petunidin are the six major anthocyanidins commonly found in fruits and vegetables [2]. This Special Issue includes 8 peer-reviewed papers (including 6 original research articles) which highlight the diverse bioactivities of ACNs and ACN-rich foods in the protection against chronic disease.

ACNs may be beneficial for health through their well-documented effects on cellular antioxidant status and inflammation [3]; however, their underlying mechanisms of action in their protection of chronic diseases are likely complex and require further elucidation. Lee et al. [4] determined whether ACN-rich blackcurrant exerts its anti-inflammatory action through modulation of macrophage phenotypes. Blackcurrant extract (BCE) was able to attenuate lipopolysaccharide (LPS)-stimulated activation of murine macrophages, as well as to repress the pro-inflammatory M1 polarization of mouse bone marrow-derived macrophages (BMDM) and human THP-1 cells. Since BCE metabolites may also display significant anti-inflammatory activities, they next treated BMDM with serum obtained from lean or obese mice fed whole freeze-dried blackcurrant. Serum from obese mice fed blackcurrant tended to reduce the expression of interleukin-6 in BMDM, but in general, serum from blackcurrant fed lean- and obese-animals did not affect M1 macrophage marker expression in BMDM. Overall, Lee et al. [4] report a novel mechanism of action of blackcurrant in suppressing macrophage pro-inflammatory responses and of BCE in inhibiting M1 macrophage polarization.

Recent meta-analyses of prospective cohort studies have reported anthocyanidin intakes were inversely associated with the risk of cardiovascular disease (CVD) [5,6] and type 2 diabetes mellitus (T2DM) [7], suggesting cardiometabolic benefits of ACN intake. Nicolae Manolescu et al. [8] reviewed studies on the health benefits associated with ACN consumption in stroke prevention, presenting data from epidemiological, in vitro, in vivo, and clinical studies. They provide evidence for beneficial effects of ACNs and their metabolites in the protection of vascular endothelium, which, through direct and indirect mechanisms, may limit stroke pathophysiology in the brain. Other vascular benefits of ACNs related to blood pressure are highlighted by Vendrame et al. [9]. They reviewed 66 human intervention trials testing the effects of purified ACNs or ACN-rich food sources on blood pressure. They found that a number of studies report significant blood pressure-lowering activity related to ACNs and ACN-rich berry consumption. However, about half of the studies reviewed did not observe blood pressure-lowering effects, which could be due to several factors, such as dosage and/or duration, food matrix effects, as well as baseline blood pressure and ACN intake of the population. Espinosa-Moncada et al. [10] report the effects of *Vaccinium meridionale* Swartz (known as “agraz”), a blueberry from Colombia, in individuals with metabolic syndrome (MetS), a population who are at elevated risk for CVD and T2DM. In a clinical study, they evaluated the effects of 200 mL of agraz nectar or placebo consumption on inflammatory and oxidative stress markers, in a 4-week double blind, crossover study in women with MetS. Although most characteristics of MetS were unchanged with agraz, the berry altered several markers related to antioxidant status. Notably, serum antioxidant capacity measured by the 2,2-diphenyl-1-picrylhydrazyl (DPPH) method was increased, while urinary 8-hydroxy-2′-deoxyguanosine (8-OHdG) was lower after agraz consumption, compared to the placebo. These results suggest that phenol-rich agraz consumption may counteract oxidative stress in those with MetS. 

Recently, the important roles of ACN intake on modulating gut microbiota and gut health have emerged. Evidence suggests that health benefits conferred by ACN intake are partly dependent on gut microbiota [11]. Kilua et al. [12] studied the effects of purple sweet potato polyphenols (PSP) on a mixed culture of swine fecal bacteria during in vitro colonic fermentation. They showed that there were interactions between PSP and fiber type (cellulose, inulin) on microbial composition, pH, and putrefactive product levels. Most notably, PSP lowered pH, increased *Bifidobacterium* and *Lactobacillus* relative abundances, and lowered levels of *p*-cresol (a putrefactive product) when combined with cellulose fiber. In combination with inulin, PSP increased *Lactobacillus*, yet lowered *Bifidobacterium* relative abundance. Thus, PSP may alter the microbial composition differently depending on the fermentability of fiber in the diet, although in vivo research is needed to confirm this. Wankhade et al. [13] explored a possible sex-dependent modulation of the gut microbiota following a 4-week blueberry supplementation of C57BL/6J mice. Supplementation of a modified AIN-93G diet (17% kcal as fat) with 5% (*w*/*w*) freeze-dried blueberry powder modulated cecal microbial communities, as seen by decreased α-diversity, a lower Firmicutes/Bacteroidetes ratio, and altered β-diversity after 4 weeks. Sexually-dimorphic responses to blueberry feeding were observed at the genus level, where 16 genera (5 unique) in male mice and 21 genera (10 unique) in females were significantly affected. Additionally, predicted metagenomics showed fatty acid and lipid metabolism pathways were associated with specific microbes only in male mice. Studies in humans are warranted to confirm such interactions between ACNs and/or blueberry components, sex, and the gut microbiome. 

Beyond indirect effects on host health via modulating the gut microbiome, ACNs may directly influence gut health via the immune cells found in gut-associated lymphoid tissue. Pei et al. [14] evaluated whether aronia berry consumption could inhibit colitis by modulating the antioxidant function of immune cells and gastrointestinal tissues. To induce colitis in mice, they adoptively transferred naïve T cells into recombinase activating gene-1 deficient (*Rag1*^−/−^) animals. They then fed mice with either a 4.5% (*w*/*w*) aronia berry-supplemented diet or a control diet for 5 weeks. Aronia berry was found to attenuate colitis (lower colon weight/length ratios, 2-deoxy-2-[^18^F]fluoro-d-glucose uptake, and pro-inflammatory gene expression), while preventing colitis-associated oxidative damage and depletion of antioxidants in colon tissue. Aronia berry also affected lymphoid tissues, as it was shown to upregulate expression of anti-inflammatory interleukin-10 and antioxidant enzymes in mesenteric lymph nodes and to lower splenic mitochondrial H_2_O_2_ production. Thus, aronia berry attenuated colitis via boosting antioxidant status in colon and lymphoid tissues.

There is emerging evidence of beneficial effects on bone health with the consumption of ACNs [15,16]. Sakaki et al. [17] evaluated whether ACN-rich BCE can improve bone mass in a mouse model of age-related bone loss. In this study, the investigators fed either a standard chow diet or a chow diet with 1% (*w/w*) BCE for 4 months to female C57BL/6J mice, aged 3 months old (young; *n* = 20) and 18 months old (aged; *n* = 15). BCE supplementation was found to benefit several markers of bone health in both young and aged mice. In young mice, BCE increased the trabecular bone volume fraction as well as glutathione peroxidase activity in bone homogenate, and lowered a marker of bone resorption. In aged mice, BCE increased the osteoblast surface, tended to increase bone mineral content, catalase activity, and lowered tumor necrosis factor-α concentrations in bone homogenate. Overall, the findings from Sakaki et al. suggest that early consumption of ACN-rich BCE may protect against aging-associated bone loss. This may be related to an enhancement in antioxidant defenses in bone, although the exact mechanism is unclear. It is worth noting that a strength of this study was the use of an age-related model of bone loss, instead of the often-studied ovariectomized (OVX)-model of bone loss, which mimics postmenopausal bone loss and not aging-related bone loss.

In conclusion, this Special Issue contributes important findings related to the health benefits ACNs and ACN-rich foods. As a guest editor, I would like to thank the authors for their contributions to this Special Issue and acknowledge the reviewers for their valuable feedback throughout this process. 
